# Debt-free cardiac health

**DOI:** 10.1186/1532-429X-18-S1-P247

**Published:** 2016-01-27

**Authors:** Victor Farah, Mark Doyle, Geetha Rayarao, Diane V Thompson, Ronald B Williams, June A Yamrozik, Moneal Shah, Robert W Biederman

**Affiliations:** Cardiac MRI, Allegheny General Hospital, Pittsburgh, PA USA

## Background

Predicting cardiac prognosis and outcome is important. As the heart progresses towards failure, it is known that the ventricular-vascular coupling (VVC) progresses from the optimal range (0.5 to 1.2) to values as high as 10 in severe failure. However, even hearts close to failure may exhibit a VVC in the normal range and thus its future predictive value is low. Here we consider the cardiac energy usage derived from cardiovascular magnetic resonance (CMR) to better utilize VVC data. To accomplish this we consider the difference between internal energy (E_Internal_) and external energy (E_External_) which we term energy debt (E_Debt_).

## Objective

To establish the relationship between E_Debt_ and VVC and show that E_Debt_ adds to the assessment of cardiac health.

## Methods

**Figure 1:**
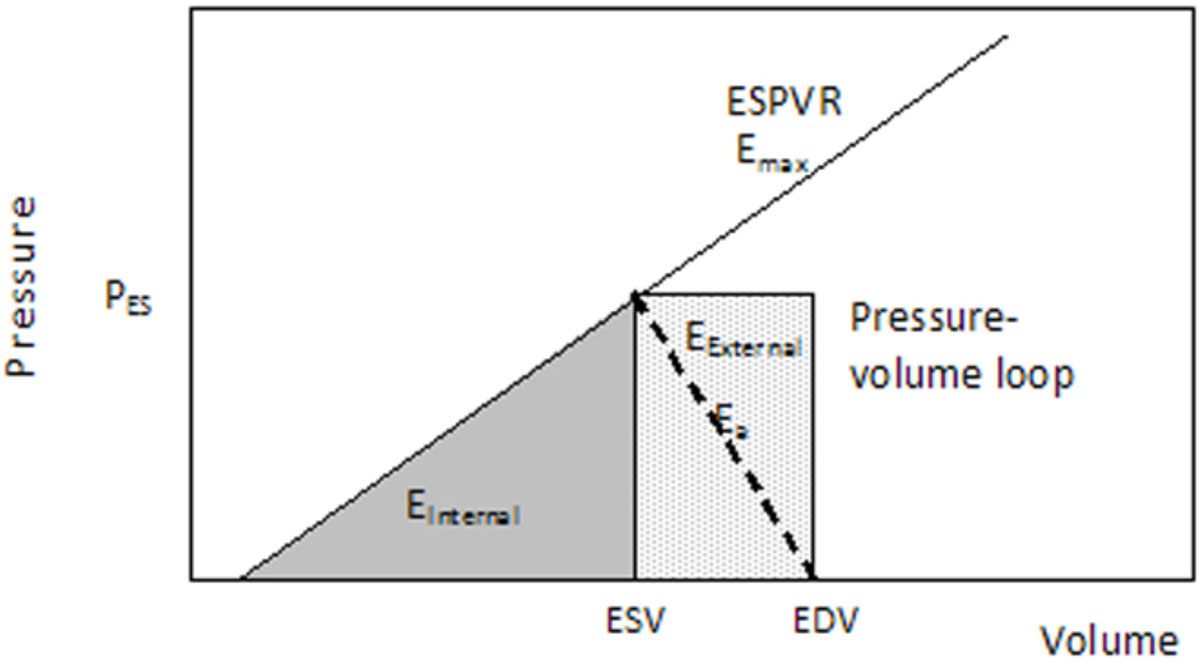
Figure 1

CMR volumetric image data were collected in patients (n = 90) undergoing functional evaluation to measure; end-systolic volume (ESV), end-diastolic volume (EDV), stroke volume (SV), blood pressure and heart rate. From the end-systolic pressure volume relationship (ESPVR) (Figure 1) the E_Internal_ is calculated as:

E_Internal_ = ½ ESV.Pes (equ 1)

Where P_es_ is considered to be approximated to the mean arterial pressure:

MAP = [DBP + 1/3 (SBP-DBP)] (equ 2)

The E_External_ is calculated as:

E_External_ = SV x P_es_ (equ 3)

VVC is calculated as:

VVC = ESV/SV (equ 4)

E_Debt_ is calculated as:

E_Debt_ = E_Internal_ - E_External_ (equ 5)

By substitution and arrangement:

E_Debt_ = ½ P_es_ x SV x (VVC-2) (equ 6)

## Results

**Figure 2:**
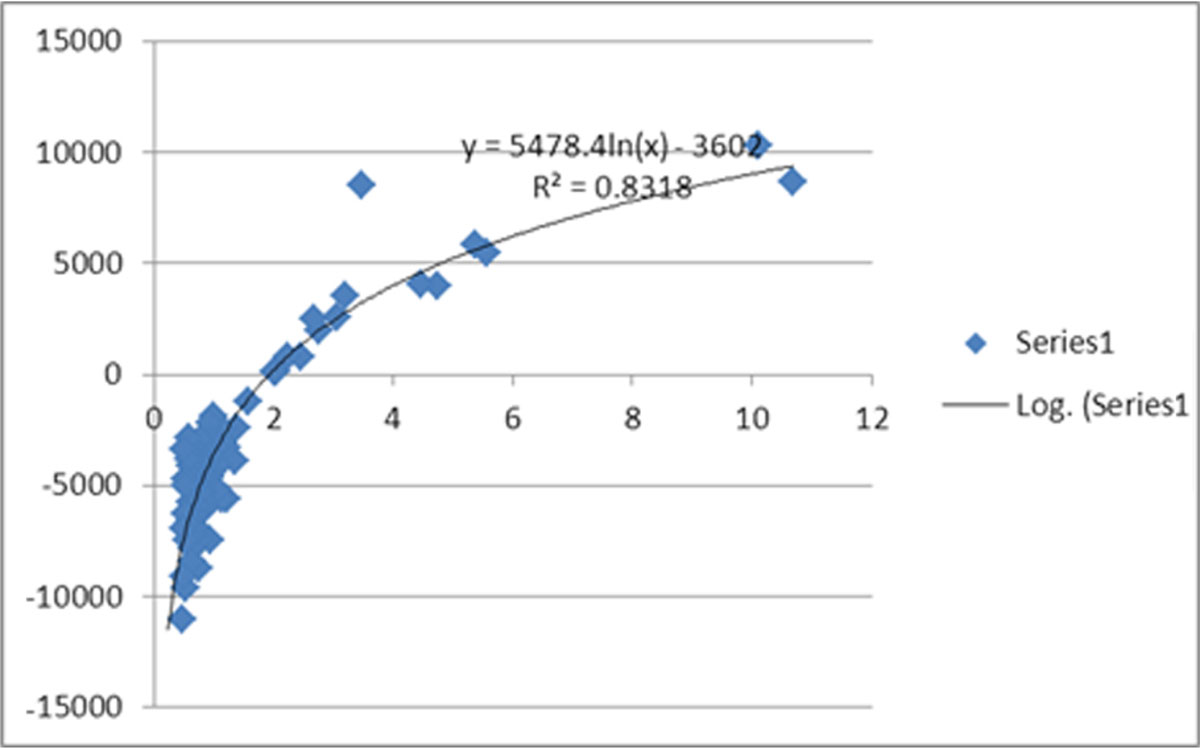
Figure 2

The plot of VVC vs. E_Debt_ is fitted to a log curve (Figure 2). As is apparent from equ 6 and Figure 2, when the E_Debt_ transitions from positive to negative the VVC exceeds 2. Further, note the high steepness of the curve for values when E_Debt_ is positive.

## Conclusions

Here we consider the net difference between internal and external ventricular work derived from non-invasive PV loops acquired during routine CMR exams. By regarding internal work as a *negative* burden and external work as a *positive* expression of cardiac function, we show that hearts with a net energy debt cross over to failure, while hearts with a net positive energy expression function normally. Thus, E_Debt_ provides additional information, since even when the VVC is within the optimal working range, the heart may be close to crossing the debt line, masquerading dysfunction. Once crossed, the curve indicates that rapid acceleration to a high VVC may imminently follow. Importantly, we show that even hearts with a healthy VVC value, heretofore believed to be advantageous, may be close to failure by the net energy debt criteria.

